# Supplementary material for: The Diverse Calpain Family in Trypanosomatidae: Functional Proteins Devoid of Proteolytic Activity?
Source: Cells. 2021 Feb 1;10(2):299. doi: 10.3390/cells10020299 (PMC7912814; doi:10.3390/cells10020299)
Supplement: Supplementary file 1 [file cells-10-00299-s001.pdf]

cells MDPI

Supplemantary Information

The Diverse Calpain Family in Trypanosomatidae: Functional Proteins Devoid of Proteolytic Activity?

Table 1. Calpain sequences retrieved from *A. deanei* genome.

Sequence ID	Assemble	Domain architecture	Predicted molecular mass (kDa)	Conserved catalytic triad (C,H,N)	Note
**EPY23292.1**<br>**CAD2213457.1**	GCA_000442575.2<br>GCA_903995115.1	DUF1935	14.54	------	Identical Proteins
**EPY28159.1**<br>**CAD2219314.1**<br>**CAD2219315.1**	GCA_000442575.2<br>GCA_903995115.1<br>GCA_903995115.1	DUF1935	12.92	------	Identical Proteins
**EPY33088.1**<br>**CAD2215177.1**	GCA_000442575.2<br>GCA_903995115.1	DUF1935	15.25	------	Identical Proteins
**EPY34498.1**<br>**EPY43070.1**<br>**EPY43802.1**<br>**CAD2215188.1**	GCA_000442575.2<br>GCA_903995115.1	DUF1935	16.09	------	Identical Proteins
**EPY37700.1**<br>**CAD2215183.1**	GCA_000442575.2<br>GCA_903995115.1	DUF1935	20.03	------	Identical Proteins
**EPY38860.1**<br>**EPY39796.1**<br>**CAD2215173.1**	GCA_000442575.2<br>GCA_903995115.1	DUF1935	14.39	------	Identical Proteins
**EPY41474.1**<br>**CAD2215168.1**	GCA_000442575.2<br>GCA_903995115.1	DUF1935	14.06	------	Identical Proteins
**EPY42018.1**	GCA_000442575.2	DUF1935	15.82	------	------
**EPY38340.1**	GCA_000442575.2	fragmented CysPc	23.97	------	Short amino acid sequence from the catalytic domain
**EPY38663.1**	GCA_000442575.2	fragmented CysPc, CBSW	32.88	------	Short amino acid sequence from the catalytic domain
**EPY17791.1**<br>**CAD2215189.1**	GCA_000442575.2<br>GCA_903995115.1	CysPc	80.68	------	Identical Proteins
**EPY31064.1**	GCA_000442575.2	CysPc	61.9	present	Identical Proteins

**CAD2217327.1**	GCA_903995115.1				
**EPY33036.1**	GCA_000442575.2	CysPc	53.67	------	------
**EPY19782.1**<br>**CAD2214161.1**	GCA_000442575.2<br>GCA_903995115.1	CysPc	104.13	------	Identical Proteins
**EPY35501.1**<br>**CAD2215192.1**	GCA_000442575.2<br>GCA_903995115.1	CysPc	41.89	------	Identical Proteins
**CAD2215193.1**	GCA_903995115.1	CysPc	56.70	------	------
**EPY40913.1**<br>**CAD2215397.1**	GCA_000442575.2<br>GCA_903995115.1	CysPc	30.53	------	Identical Proteins
**CAD2215575.1**	GCA_903995115.1	CysPc	75.57	------	------
**EPY17550.1**<br>**CAD2222307.1**	GCA_000442575.2<br>GCA_903995115.1	CysPc	54.99	------	Identical Proteins
**EPY40095.1**	GCA_000442575.2	CysPc	66.74	present	------
**EPY16971.1**	GCA_000442575.2	2xCysPc	50.11	------	------
**EPY35473.1**	GCA_000442575.2	DUF1935, CysPc	81.01	present	------
**EPY34508.1**<br>**EPY37930.1**<br>**CAD2214023.1**	GCA_000442575.2<br>GCA_903995115.1	DUF1935, CysPc	95.30	------	Identical Proteins
**EPY27435.1**<br>**CAD2214024.1**	GCA_000442575.2<br>GCA_903995115.1	DUF1935, CysPc	95.86	------	Identical Proteins
**EPY24669.1**<br>**CAD2214025.1**	GCA_000442575.2<br>GCA_903995115.1	DUF1935, CysPc	82.47	------	Identical Proteins
**EPY28634.1**<br>**CAD2214026.1**	GCA_000442575.2<br>GCA_903995115.1	DUF1935, CysPc	128.39	------	Identical Proteins
**EPY39328.1**<br>**CAD2214027.1**	GCA_000442575.2<br>GCA_903995115.1	DUF1935, CysPc	88.95	------	Identical Proteins
**CAD2215190.1**	GCA_903995115.1	DUF1935, CysPc	77.06	------	------
**EPY26598.1**<br>**EPY43876.1**<br>**CAD2215191.1**	GCA_000442575.2<br>GCA_903995115.1	DUF1935, CysPc	77.53	------	Identical Proteins

**CAD2215195.1**	GCA_903995115.1	DUF1935, CysPc	77.01	------	------
**EPY26455.1**	GCA_000442575.2	DUF1935, CysPc	77.03	------	------
**EPY27608.1**<br>**CAD2220197.1**	GCA_000442575.2<br>GCA_903995115.1	DUF1935, CysPc	94.24	present	Identical Proteins
**EPY29841.1**	GCA_000442575.2	DUF1935, CysPc	80.85	------	------
**EPY31384.1**	GCA_000442575.2	DUF1935, CysPc	46.69	------	------
**EPY32139.1**<br>**CAD2214018.1**	GCA_000442575.2<br>GCA_903995115.1	DUF1935, CysPc	80.20	present	Identical Proteins
**EPY35155.1**	GCA_000442575.2	DUF1935, CysPc	75,67	------	------
**EPY35937.1**	GCA_000442575.2	DUF1935, CysPc	80.33	present	------
**EPY36766.1**	GCA_000442575.2	DUF1935, CysPc	62.07	------	------
**EPY37455.1**	GCA_000442575.2	DUF1935, CysPc	85.88	------	------
**EPY40346.1**	GCA_000442575.2	DUF1935, CysPc	77.54	------	------
**EPY41899.1**	GCA_000442575.2	DUF1935, CysPc	83.53	------	------
**EPY21044.1**<br>**CAD2218077.1**	GCA_000442575.2<br>GCA_903995115.1	CysPc, CBSW	73.48	------	Identical Proteins
**EPY41780.1**<br>**CAD2216952.1**	GCA_000442575.2<br>GCA_903995115.1	CysPc, CBSW	123.76	present	Identical Proteins
**CAD2220901.1**	GCA_903995115.1	CysPc, CBSW	121.94	------	------
**CAD2213168.1**	GCA_903995115.1	CysPc, CBSW	130.64	------	------
**EPY20406.1**	GCA_000442575.2	CysPc, CBSW, CysPc	217.36	------	------
**EPY22262.1**	GCA_000442575.2	CysPc, CBSW, CysPc	174.58	------	------
**EPY32574.1**	GCA_000442575.2	CysPc, CBSW, CysPc	98.51	------	------
**CAD2213171.1**	GCA_903995115.1	CysPc, CBSW, CysPc	97.73	------	------
**CAD2215402.1**	GCA_903995115.1	CysPc, CBSW, CysPc	354.59	------	------
**EPY22707.1**<br>**CAD2215572.1**	GCA_000442575.2<br>GCA_903995115.1	RPT1, CysPc, CBSW	236.37	------	Identical Proteins
**CAD2215393.1**	GCA_903995115.1	CysPc, CBSW, CysPc, RPT1	522.62	------	------
**EPY28067.1**<br>**CAD2221641.1**	GCA_000442575.2<br>GCA_903995115.1	KISC, 3xARM, CBSW	103.81	------	Identical Proteins; Kinesin motor domain associated with CBSW

**CAD2215401.1**	GCA_903995115.1	RPT1, CysPc	189.04	------	------
**EPY20404.1**	GCA_000442575.2	2xRPT1, CysPc	161.06	------	------
**CAD2215396.1**	GCA_903995115.1	RPT1, RPT2, CysPc	301.59	------	------
**EPY21560.1**	GCA_000442575.2	2xRPT1	24.58	------	RPT sequences annotated as calpain-like proteins
**EPY27093.1** **CAD2215574.1**	GCA_000442575.2 GCA_903995115.1	2xRPT1	157.43	------	Identical Proteins; RPT sequences annotated as calpain-like proteins
**EPY16212.1** **CAD2215398.1**	GCA_000442575.2 GCA_903995115.1	2xRPT1	11.23	------	Identical Proteins; RPT sequences anotated as calpain-like proteins
**EPY20405.1**	GCA_000442575.2	2xRPT1	58.08	------	RPT sequences anotated as calpain-like proteins
**EPY22264.1**	GCA_000442575.2	2xRPT1	125.40	------	RPT sequences anotated as calpain-like proteins
**EPY24573.1**	GCA_000442575.2	2xRPT1	239.87	------	RPT sequences anotated as calpain-like proteins
**EPY25974.1** **CAD2215400.1**	GCA_000442575.2 GCA_903995115.1	2xRPT1	189.50	------	Identical Proteins; RPT sequences anotated as calpain-like proteins
**EPY29704.1**	GCA_000442575.2	RPT1, RPT2, RPT1	101.27	------	RPT sequences anotated as calpain-like proteins

The ID of the calpain sequences was retrieved from two different assemblies of the *Angomonas deanei* genome (Genebank ID 14191). The sequences were ordered based on their domain architecture. The predicted molecular mass was calculated in http://www.bioinformatics.org/sms/prot_mw.html. Sequences annotated as “calpain-like proteins” but with less than 100 amino acids or without any calpain associated domain were not included. CBSW—calpain-type alpha-beta-sandwich domain, CysPc—proteolytic core domain, KISC—kinesin domain, DUF1935—conserved N-terminal calpain-related domain from trypanosomatids, RPT—repeated domain found in de-ubiquitinating proteins, 2× and 3× indicate the number of times that a domain appears in the sequence. Fragmented CysPc stands for short amino acid sequences from the catalytic domain.

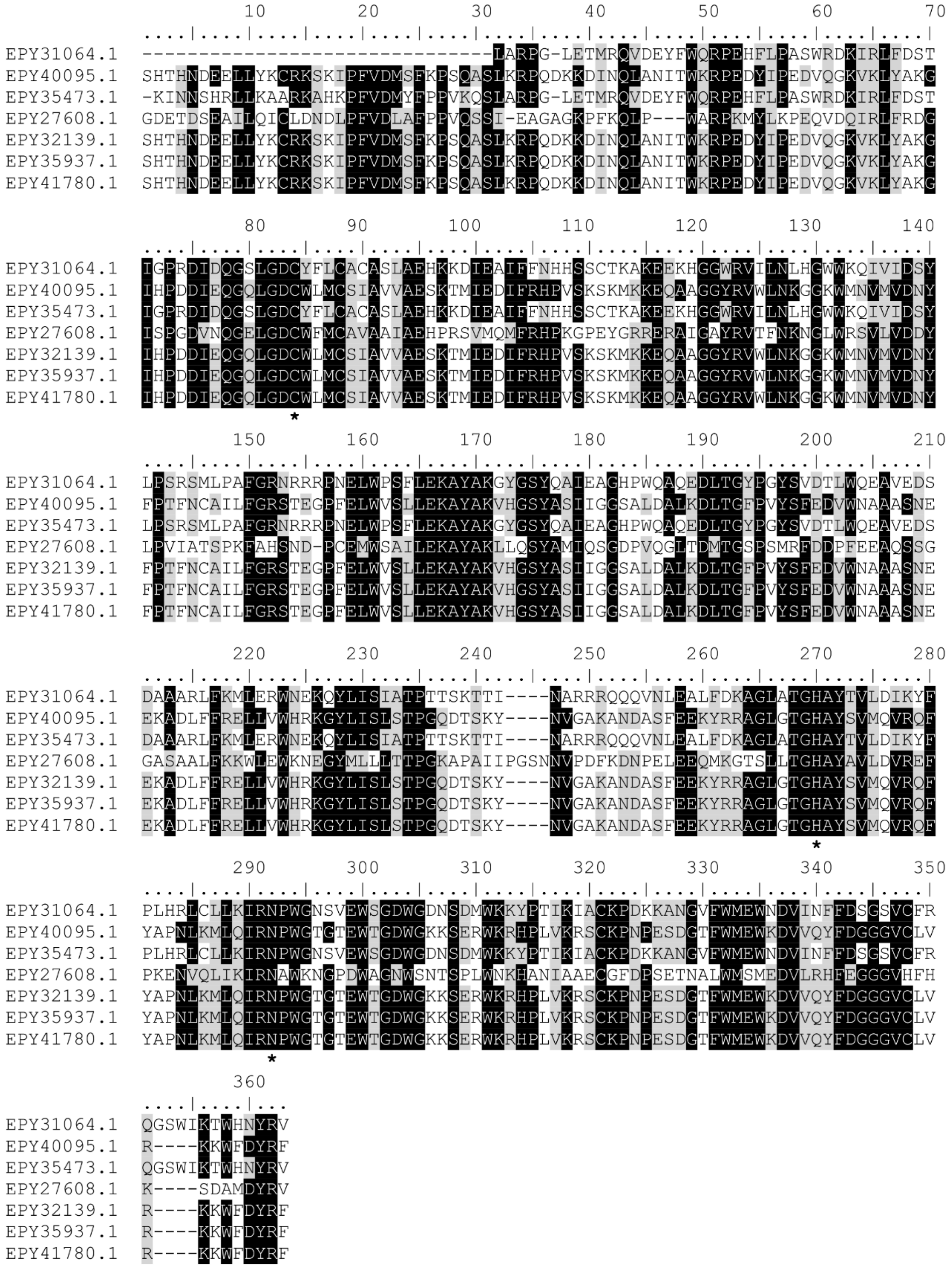

Figure S1. Multiple sequence alignment of the cysteine peptidase core (CysPc) domain of *Angomonas deanei* with the conserved catalytic triad (C,H,N). The sequence alignment was performed using ClustalW. GeneDB ID of the *A. deanei* calpain sequences are on the left. Identical and similar residues are represented in black and grey, respectively. The amino acids responsible for the proteolytic activity of conventional calpains are indicated by a *.
